# The Tooth Brush as a Prophylactic

**Published:** 1876-01

**Authors:** Abr. Robertson


### THE TOOTH BRUSH AS A PROPHYLACTIC.

BY ABR. ROBERTSON, M. D., D. D. S.

*Mr. President and members of the Merrimac Valley Dental Society:*—By request of your Executive Committee I propose to give you, on this occasion, a short essay on a small subject, on which I am well aware that I take the unpopular side. My subject is, The tooth brush as a prophylactic. It is a trite saying, and as true now as it was when it was first uttered, that "cleanliness is near akin to godliness," and it has seemed so true that it has also been said, that "no man will do a mean act in a clean shirt."

A due regard for cleanliness of person, conduces, no doubt, to health, comfort and comeliness; while a wanton neglect of it, deteriorates comeliness, comfort and health, and is moreover, a cause of chagrin and disgust to those more cleanly, with whom they associate or come in contact.

But, as man is neither an aquatic nor an amphibious animal, washing and bathing can be and sometimes are, carried to excess.

It is a matter of quite common observation, that those who bathe their whole person every day, after a time, become apparently bloodless and greatly debilitated.

The habits of civilization as a matter of cleanliness and comfort, make the use of the tooth brush a necessity. If we followed the teachings of our instincts, if we allowed our reason to lead us to the observance of the laws of nature, in our diet and domicile, as the lower animals do, we should have no more use for tooth brushes or for tooth picks than they do.

No wild or even domestic animal ever suffers from decay or other disease of their teeth, when unrestrained in following their instincts in these respects.

But it is a notorious fact that horses which are highly pampered, kept on an unnatural diet, and kept in dark and ill ventilated stalls, suffer from the decay and loss of their teeth; and cows, fed on brewer's slops, for a comparatively short time, from their loosening and falling out. A caged squirrel kept but for a few months—less than six—on cake and soft fruits, will lose the power, by the loosening of its teeth, to open a nut with a shell no harder than a filbert. The last I know from personal observations.

Great stress has been, and still is, laid by almost all practitioners of dentistry—so far, at least, as I know,—on the constant and frequent use of the tooth brush; and often with the use of it, tooth powders containing pulverized pumice stone or other gritty substances, and especially, as a prophylactic or preventive of decay.

A few years ago, a very eminent and influential dentist, in one of our largest western cities, published the statement that "all dentists very well knew that perfect cleanliness was a sure preventive of decay of the teeth." At that time having been full twenty years occupied in looking after and caring for the preservation of human teeth; I did not know or believe the statement to be correct or founded on fact, neither do I now believe or know it.

Contrary to, and in despite of all these, as I think vague assertions, I now, after long and varied experience and observation, but not without care and due consideration, presume to say and shall undertake or try to prove, that the tooth brush, as a prophylactic, has but very little if any beneficial influence in any case, and nearly if not in all cases, none at all.

The truth of this statement, and my attempt at proving it, I shall base upon two distinct grounds.

First, after an experience in the constant and active practice of dentistry, for about thirty years, I have frequently noticed, as many others also have, that persons who have taken the utmost possible care for the cleanliness of their teeth by brushing them soon after every time of eating and just before going to bed, have suffered more from the decay of their teeth, and have lost them sooner than many others, who never used a tooth brush or took any sort of care for or about their teeth.

The history of my own denture is a marked illustration of the verity of this statement.

I am now more than sixty-three years of age, have not a decayed tooth in my mouth, have all the permanent teeth I ever had except three; have never had but one tooth filled, and that not by human hands; but with her ever busy fingers old dame nature filled it herself; and not with gold or any amalgamated alloy, or Hill's stopping, or chloride of zinc, or by any other artificial material, but with her own make of the natural material, dentine.

Of this, more anon, I never used a tooth brush or took any other means for cleansing or preserving my teeth, till I was twenty-eight years old. Indeed I had never seen but a few tooth brushes till nearly that time; and I remember of knowing but one man, but a short time before that, who constantly used one; and I think he used to be laughed about for using it.

I have already said that I had lost but three of my permanent teeth and these were not from decay. Although it may be an unwarranted digression, I will venture to say, that my first lost was my first upper right molar; this had a small imperfection in the enamel, in its grinding surface; the defect was not from decay, but from what cause I do not know. I have others in the same or rather in a worse condition now. This tooth used frequently to be very sensitive; and when a youth of about seventeen, on eating some black berries as a dessert, I got a seed in the defective place which caused me some pain; so, without thinking in the least of the worth of a tooth, I thought it would be *fun* to have it out and went to a man who sometimes "*pulled*" teeth, and very foolishly asked him to take it out; he, without sense enough to know that he ought not to extract a good serviceable tooth, simply because a silly boy wanted it out, put on his old turn key, wrenched away and broke it off, almost to the exposing of the pulp. I did not find the expected fun, either then or when inflammation and suppuration of the pulp occurred, with a subsequent badly swelled cheek and the formation of an abscess, which took place a while after.

The other two were my lower wisdom teeth; and by the way, none of my wisdom teeth came in sight till after I was thirty years old, and as yet, I have had only three, one upper and two lower. The upper came a year or two before the lower, and it and the second molar on the other side of my mouth, had so far advanced that the lower ones had not room for their full development, that is to say, a part of their crowns were below the margin of their gums and thus formed a kind of *cul de sac*, into which food, in the process of mastication, would be forced, from whence it was quite difficult to remove, so it frequently produced irritation, inflammation and swelling of the gums, with severe pain; and as they were of no especial use, I had them extracted.

I will further digress to say, that besides the lack of one wisdom tooth, I have never had as many teeth by two, as is the rule or as almost every one else has. My upper lateral incisors are wanting; never had any; this defect in my dentine is hereditary; by various observations I have learned that I inherited it from my great grandfather; who was the father of my father's mother.

I found the same defect in several of my second cousins, as well as in others of my own father's family.

In most of the instances, the upper lateral incisors were entirely wanting; while in a few cases there were rudimentary teeth only, sometimes but one and sometimes both, which were round and about the size, length and sharpness of a cat's cuspid tooth.

My second argument against the tooth brush as a prophylactic, is in the well known fact, that in a vast majority of decayed teeth the caries is found in their approximal surfaces and in the pits between the cusps of the bicuspid and multicuspid, or molar teeth, where the tooth brush can not by any possibility reach.

It is true that a great many teeth, especially in dyspeptics, where the secretion by the mouth is strongly acid, decay at, or a little under the margins of the gums; in these cases, a free use of the tooth brush, simply removes the decomposed matter, already saturated with acid, and exposes a fresh surface to be acted upon with greater avidity, thus hastening the decomposition, decay and loss of the teeth.

By all this I do not intend to denounce or ignore the judicious use of the tooth brush.

For as, I said in the beginning, the habits of civilization, our unnatural modes of living, by sometimes eating too much, by eating too great a variety of articles at a time, by the too free use of stinulating condiments, by too much stimulating drink, and by the unscientific preparation of food or ill-ventilated and darkened houses, the natural functions of our systems are deranged and the secretions changed, producing, especially, on rising in the morning, an unpleasant taste in the mouth; bad odors in the breath; and unsightly desposits on the teeth; and therefore the use of the tooth brush becomes a necessity, for promotion of health, comfort and comeliness. For these purposes let us advise its use, but let us cease stultifying ourselves by recommending it, as a sure prophylactic against decay of the teeth.

Although not altogether relevant to the subject in hand, I will say something of the anomalous case, in my own mouth, to which I have referred, where a badly decayed tooth was restored by the process of nature. I call it anomalous because I have never heard of or seen another case like it.

I will premise by saying, that my temporary right upper incisor was imperfectly formed; the dentine of its crown being nearly all wanting. This I ascertained by passing a pretty large pin up through the foramen in its root, which passed without obstruction almost to its cutting edge.

As tradition tells me, when I was about two years old, suppuration of the pulp occurred, which developed into an abscess, broke in the antrum maxilare and burst the upper part of that cavity; removing a considerable part of the lower arch of the orbit, the effect of which was distinctly visible, till I had nearly attained my growth and for some of the first years, caused a slight deformity. From this, a derangement of the surrounding parts probably occurred, and from this or from some other cause unknown, its successor, the permanent incisor was imperfectly formed; so it was too late in coming, a year or more later than its mate.

Before one half the length of the crown had protruded through the gum, it proved to be decayed, the caries extended entirely across the labial surface, from very near the cutting edge, to a sixteenth of an inch or more, in width, and of very considerable depth, with the usual dark color accompanying carious teeth. This continued with but little material change for several years. But before I had arrived at manhood, a deposit of dentine had been made, almost filling the cavity, entirely indeed, except some slight corrugations on its sides where it came in contact with the enamel.

Many long years of constant use, as usually occurs, has worn away a portion of the tooth, enough to have removed fully one half, I think, of the new deposit. But there is still enough of it remaining to be distinctly seen, and to verify the statement as any one here may see who wishes.

As I have said, this is an anomalous case, the like of which I have never seen or heard, and I do not believe that this would have occurred if I had disturbed the processes of nature by using a tooth brush.

